# Insulin Resistance-related Gray Matter Volume Reduction is Associated with the Default Mode Network

**DOI:** 10.14789/ejmj.JMJ24-0044-OT

**Published:** 2025-01-30

**Authors:** SAKI ASANO, AKITOSHI OGAWA, TAKAHIRO OSADA, SATOSHI OKA, KOJI NAKAJIMA, YASUSHI OSHIMA, SAKAE TANAKA, HIDEYOSHI KAGA, YOSHIFUMI TAMURA, HIROTAKA WATADA, RYUZO KAWAMORI, SEIKI KONISHI

**Affiliations:** 1 Department of Neurophysiology, Juntendo University School of Medicine, Tokyo, Japan; 1 Department of Neurophysiology, Juntendo University School of Medicine, Tokyo, Japan; 2 Department of Orthopaedic Surgery, The University of Tokyo School of Medicine, Tokyo, Japan; 2 Department of Orthopaedic Surgery, The University of Tokyo School of Medicine, Tokyo, Japan; 3 Department of Metabolism and Endocrinology, Juntendo University School of Medicine, Tokyo, Japan; 3 Department of Metabolism and Endocrinology, Juntendo University School of Medicine, Tokyo, Japan; 4 Sportology Center, Juntendo University School of Medicine, Tokyo, Japan; 4 Sportology Center, Juntendo University School of Medicine, Tokyo, Japan; 5 Research Institute for Diseases of Old Age, Juntendo University School of Medicine, Tokyo, Japan; 5 Research Institute for Diseases of Old Age, Juntendo University School of Medicine, Tokyo, Japan; 6 Advanced Research Institute for Health Science, Juntendo University School of Medicine, Tokyo, Japan; 6 Advanced Research Institute for Health Science, Juntendo University School of Medicine, Tokyo, Japan

**Keywords:** voxel-based morphometry, homeostatic model assessment for insulin resistance, functional connectivity, hypothalamus

## Abstract

In this study, we observed that insulin resistance is linked to a reduction in grey matter volume in the default-mode and limbic networks of the cerebral cortex in older adults. Additionally, we found that the paraventricular nucleus of the hypothalamus is significantly functionally connected to these two cortical networks. Our results suggest that the reduction in gray matter volume associated with insulin resistance arises through metabolic homeostasis mechanisms in the hypothalamus.

Insulin resistance refers to a reduced sensitivity of body tissues (such as muscle, fat, and liver) to insulin^1, 2)^. When insulin resistance increases, insulin production is no longer adequate, potentially leading to type 2 diabetes mellitus (T2DM). Brain cells, like muscle and fat cells, can become less sensitive to insulin, a condition called “brain insulin resistance.” This condition can impair synaptic, metabolic, immune response functions, and cognitive functions^3-12)^. The hypothalamus interacts with the cerebral cortex, particularly the default-mode network (DMN), as demonstrated through resting- state functional connectivity (RSFC)^13, 14)^. Studies of gray matter volume (GMV) in the brain have demonstrated its relationship with homeostasis model assessment for insulin resistance (HOMA-IR)^15)^. Here, we present our recent study investigating the association between insulin resistance and both GMV and RSFC in the human brain. Our study utilized structural images and blood samples from a cohort of over 1,000 older adults in Japan^16)^ and RSFC data from the Human Connectome Project (HCP)^17)^. We then discuss the implications of our findings.

Data from the Bunkyo Health Study^16)^ was used in our study (N = 1605, 679 men and 926 women, aged 73.1 ± 5.4 years, ranging from 65 to 84 years). Data collection spanned over two days for each participant. On the first day, a whole brain structural T1-weighted image was acquired using a 0.3-T magnetic resonance imaging (MRI) scanner (AIRIS Vento). Structural images were obtained using a three-dimensional gradient echo with inversion recovery sequence (repetition time = 25 ms; echo time = 5.8 ms; inversion time = 600 ms; flip angle = 12°; field of view = 200 × 250 × 250 mm^3^; resolution = 0.98 × 0.98 × 2.0 mm^3^).

On the second day, fasting blood and urine samples were collected. The homeostasis model assessment for insulin resistance (HOMA-IR)^15)^ was calculated from the fasting glucose and insulin levels. The acquired structural images were segmented, normalized to the Montreal Neurological Institute standard space, and smoothed (Full width at half maximum = 8 mm). Using a voxel-based morphometry (VBM) working on the SPM12 software package (www.fil.ion.ucl.ac.uk/spm/), we analyzed the relationship between GMV and HOMA-IR.

We also examined the functional connectivity of the cerebral regions and the hypothalamic nuclei. For the cerebral regions, we used the 360 cerebrocortical parcels provided by the HCP^18)^. Five hypothalamic nuclei were defined according to previous reports^19, 20)^. We analyzed the data of resting-state functional imaging data (N = 418) from Human Connectome Project Young Adult (HCP-YA)^17)^ using the HCP analysis pipelines^21)^. We used the denoised volumetric data for the hypothalamus and the cerebral surface data for the cerebrocortical parcels.

We found that the GMV reduction in the precuneus (general linear model analysis, t = 5.10, peak = [x = +2, y = -58, z = +28] in the coordinates of Montreal Neurological Institute (MNI), cluster size = 4044 voxels), superior frontal gyrus (t = 4.48, peak = [x = +20, y = +30, z = +38]_MNI_, 885 voxels), medial frontal gyrus (t = 4.37, peak = [x = -14, y = +32, z = +30]_MNI_, 1015 voxels), parietal operculum (t = 4.29, peak = [x = +63, y = -10, z = +9]_MNI_, 864 voxels), and ventromedial prefrontal cortex (t = 4.28, peak = [x = +10, y = +58, z = -21]_MNI_, 973 voxels) was negatively associated with HOMA-IR (Figure 1A). The greater the GMV, the smaller HOMA-IR. Among the seven cerebrocortical networks^22)^ (i.e., the DMN, limbic (LIMN), visual, somatomotor, ventral attention, dorsal attention, and frontoparietal networks), the DMN accounted for approximately 60% of the voxels in the detected clusters (Figure 1B).

**Figure 1:**
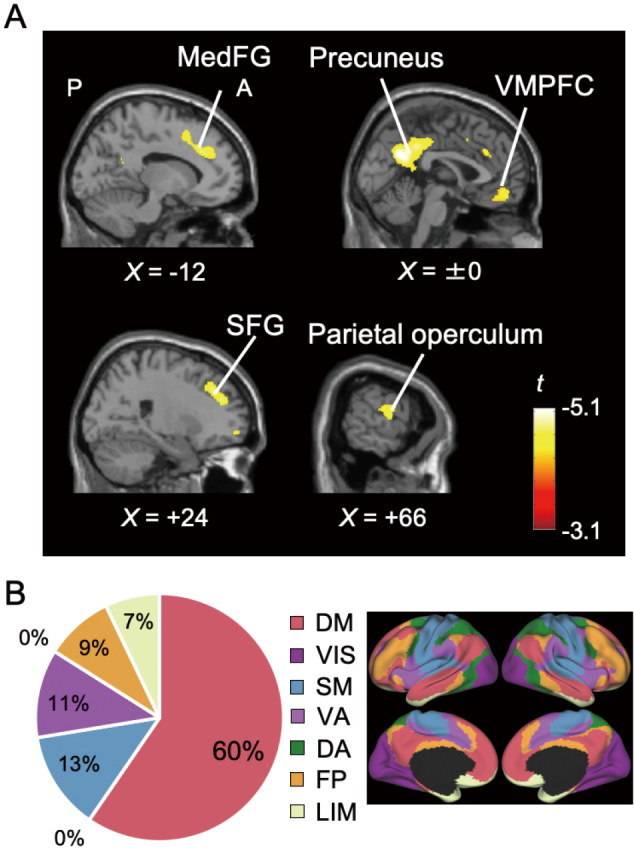
Relationship between regional GMV and HOMA-IR A. Brain regions with a significant negative correlation between regional GMV and HOMA-IR shown on sagittal sections. The largest cluster is observed in the precuneus. Other significant clusters are found in the medial frontal gyrus (MedFG), superior frontal gyrus (SFG), ventromedial prefrontal cortex (VMPFC), and parietal operculum. Labels indicate orientation: A, anterior; P, posterior; L, left; R, right. B. Distribution of voxels in significant clusters across seven cerebrocortical networks^22)^. The default-mode network is predominant within these clusters. The pie chart shows the percentage of surface vertices assigned to each cerebrocortical network (DM, default mode; VIS, visual; SM, somatomotor; VA, ventral attention; DA, dorsal attention; FP, frontoparietal; LIM, limbic).

Using the HCP dataset^17)^, we further calculated the cerebrocortical functional connectivity (i.e., Fisher-z-transformed correlation) of five hypothalamic nuclei, which are associated with glucose sensing and control of food intake in different ways: the arcuate nucleus of the hypothalamus, dorsomedial nucleus of the hypothalamus, lateral hypothalamic area, paraventricular nucleus of the hypothalamus (PVH), and ventromedial nucleus of the hypothalamus (Figure 2A). Our results indicated that the PVH was functionally connected with the cerebrocortical regions negatively correlated with HOMA-IR (Figure 2B). The connectivity of the HOMA-IR-related clusters was much higher with the PVH than with the other four nuclei (Tukey-Kramer test, all *P* < 0.001 after one-way repeated- measures analysis of variance) (Figure 2B). The PVH was functionally connected with the DMN and LIMN (Figure 2C).

**Figure 2:**
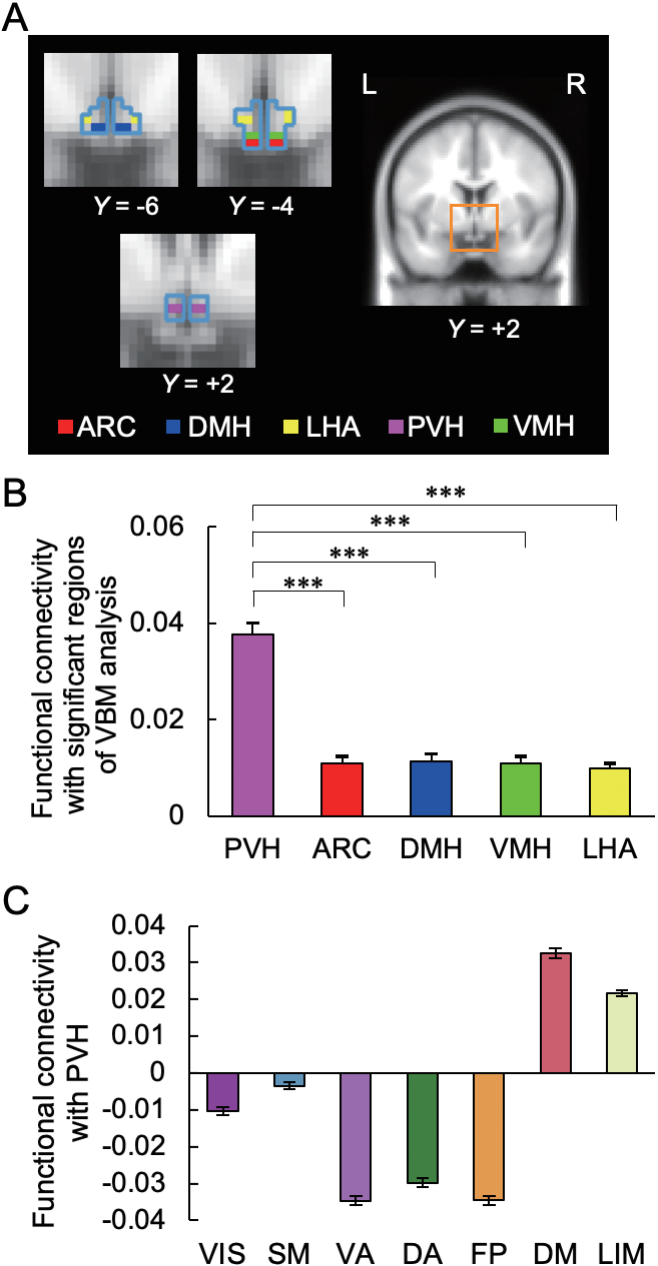
Results of functional connectivity analysis using the HCP dataset A. Regions of interests (ROIs) of hypothalamic nuclei shown on coronal sections with cyan lines indicating the border of the hypothalamus (excluding the mammillary body). L (left) and R (right) denote the hemispheres. The ROIs include the arcuate nucleus (ARC), dorsomedial nucleus of the hypothalamus, (DMH), lateral hypothalamic area (LHA), paraventricular nucleus of the hypothalamus (PVH), and ventromedial nucleus of the hypothalamus (VMH). B. Functional connectivity between the hypothalamic nuclei and the cerebrocortical parcels in the significant clusters identified in the GMV analysis. Asterisks indicate statistical significance (***P < 0.001). C. Functional connectivity between the PVH and the cerebrocortical networks. The DMN and LIMN show significantly positive connectivity with the PVH, whereas the other networks display negative connectivity.

Among brain regions, the precuneus and posterior cingulate cortex in the DMN+LIMN have the highest levels of glucose consumption^23-25)^. This finding suggests that these DMN+LIMN regions may be vulnerable to neuronal stress induced by insulin resistance. Additionally, the PVH is involved in glucose sensing^26-28)^, regulating energy expenditure^29-32)^, and controlling appetite and food intake^28, 30, 33-35)^. Based on our findings, we propose a hypothesis that insulin resistance affects glucose sensing in the hypothalamus and glucose metabolism in the regions in the DMN+LIMN through their functional connections.

## Funding

No funding was received.

## Author contributions

SA, AO, and SK designed research. SA, AO, TO, SO, KN, YO, ST, HK, YT, HW, RK, and SK performed research. SA, AO, and SK summarized data and wrote the paper.

## Conflicts of interest statement

The authors declare no competing financial interests. Yoshifumi Tamura and Seiki Konishi, who are Juntendo Medical Journal Editorial Board members, were not involved in the peer review or decision- making process for this paper.
